# Gene expression profiles in benign prostate tissue reflect tumor proximity, grade, and molecular subtype

**DOI:** 10.1186/s12967-026-09035-8

**Published:** 2026-10-03

**Authors:** Pernilla Wikström, Eva Freyhult, Julius Semenas, Marie Lundholm, Andreas Josefsson, Sofia Halin Bergström, Anders Bergh

**Affiliations:** 1 Department of Medical Biosciences, Pathology, Umeå University, Umeå, 901 85 Sweden; 2 Department of Cell and Molecular Biology, National Bioinformatics Infrastructure Sweden, Science for Life Laboratory, Uppsala University, Uppsala, Sweden; 3 Department of Diagnostics and Interventions, Urology and Andrology, Umeå University, Umeå, Sweden; 4 Wallenberg Center for Molecular Medicine, Umeå University, Umeå, Sweden

**Keywords:** Prostate cancer, Tumor adjacent histologically benign tissue, Tumor-instructed normal tissue (TINT), Transcriptomics, Tumor microenvironment, Gene expression profiling, Molecular subtype (luminal A/B), Hallmark pathways, Biomarkers

## Abstract

**Background:**

Normal tissue in a tumor-bearing organ shows morphological and molecular differences from normal tissue in a tumor-free organ. One explanation to this is that tumors actively instruct adjacent normal tissues to support growth and invasion. The aim of this study was to examine if changes in gene-expression patterns in tumor-adjacent histologically benign prostate tissue, termed tumor instructed normal tissue (TINT), were associated with tumor proximity, grade and molecular subtype and, furthermore, whether they could indicate the presence of high-grade cancer elsewhere in the prostate.

**Methods:**

Gene-expression analysis using Clariom D microarrays was performed on matched formalin-fixed paraffin-embedded samples including tumor tissue (*n* = 44), benign prostate tissue taken near (TINT-N, *n* = 44) or distant (TINT-D, *n* = 42) to the tumor in 44 men with unifocal cancer treated by prostatectomy. TINT changes related to cell proliferation and immune cell infiltration were examined using immunohistochemical markers. In addition, gene expression was compared between tumor-negative biopsies from men later diagnosed with high-grade cancer (Bx-later-tumor, *n* = 46), tumor-negative biopsies from men who remained cancer-free (Bx-control, *n* = 49), and cancer-containing biopsies (Bx-tumor, *n* = 28). Gene set enrichment analysis (GSEA) was performed to explore hallmark gene sets in tumor and TINT tissue in relation to tumor grade (high vs. low) and molecular subtype (Luminal B vs. Luminal A).

**Results:**

The transcriptome in TINT differed significantly from that in cancer-free biopsy control tissue and was related to tumor grade (high- vs. low), molecular subtype (Luminal B vs. Luminal A), and tumor proximity (TINT-N vs. TINT-D). Hallmarks related to cell proliferation and increased Ki67 staining as well as signs of increased metabolism were generally enriched in both tumor and TINT tissue of high grade and Luminal B tumors. In contrast, some immune responses were unique to TINT areas of high-grade and Luminal A tumors, respectively, and more obvious in TINT-N than in TINT-D. Analysis of Bx-later-tumor indicated enriched metabolism compared to Bx-control, without clear proliferative or immunological differences.

**Conclusions:**

TINT effects involving proliferation, metabolism and immune responses are related to distance, tumor grade and molecular subtype. TINT alterations hold potential as diagnostic and prognostic biomarkers and may provide a basis for further exploration of tumor-host interactions and potential targets for intervention.

**Supplementary Information:**

The online version contains supplementary material available at https://doi.org/10.1186/s12967-026-09035-8.

## Background

Extensive evidence shows that benign tissue in a tumor-bearing organ differs molecularly and morphologically from tissue in a tumor-free organ [1–4]. The most widely accepted explanation is the “field cancerization” hypothesis [3, 5], which proposes that large areas of an organ are altered by carcinogenic exposure, leading to precancerous genetic changes in epithelial cells and, eventually, localized tumor formation.

An alternative, though not mutually exclusive, explanation is that tumors actively instruct adjacent normal tissues to support invasion, immune evasion, and dissemination [1, 2, 6, 7]. We previously explored this concept by implanting rat prostate tumor cells of varying aggressiveness and metastatic potential into the prostates of normal immunocompetent syngeneic rats, and found evidence for morphological and molecular modifications, by us termed tumor-instructed normal tissue (TINT), in both the epithelium and stroma of the non-tumor parts of the prostate. The extent and nature of these modifications depended on tumor proximity, size, aggressiveness, and metastatic potential [2, 6–13].

In humans, similar morphological changes in benign tinted prostate tissue have been linked to tumor size, grade, and patient outcomes [8, 10–17]. The TINT concept postulates that aggressive tumors induce stronger, more extensive, and distinct effects on the surrounding tissue than less aggressive ones [2, 7, 11]. We therefore proposed that mechanisms driving TINT changes could serve as novel diagnostic and prognostic markers as well as therapeutic targets [2].

Findings by other groups support this view. In human prostates, transcriptomic profiles of tumor-adjacent benign tissue differ from those in tumor-free individuals [1, 18–22]. Aran et al. analyzed gene expression in histologically benign tissue adjacent to tumors across eight organs, including the prostate, and found that tumor-adjacent tissues exhibited expression patterns distinct from cancer-free organs, more similar to the tumor than to normal tissue [1]. This pattern, consistent across multiple organs, suggests a common cancer-associated response partly resembling that in wounding, tissue repair and healing [1, 2, 18, 23]. Some studies have also reported associations between transcriptomic profiles of benign prostate tissue and patient outcome [24, 25].

However, previous studies of benign tissue in tumor-bearing human prostates have rarely analyzed transcriptomic changes in relation to tumor grade, aggressiveness, proximity, molecular subtype, or the multifocal nature of the disease [1, 18, 20, 21, 23, 26]. Because prostate cancer is typically multifocal, with different tumors potentially influencing their surroundings differently [11], we studied a cohort of men with unifocal prostate cancer of varying grades treated by radical prostatectomy.

The aim of this exploratory study was to determine whether transcriptomic patterns in tumor-adjacent histologically benign prostate tissue (referred to as TINT) are associated with tumor proximity and histological grade, and to compare changes in gene-expression in TINT in patients to those that can be induced in an experimental model where cancer cells are implanted into a previously normal prostate. Given that prostate cancers can also be classified into molecular subtypes, Luminal A (LumA), Luminal B (LumB), and Basal, which provide prognostic information in addition to tumor grade (with LumB being associated with the poorest and LumA with the most favorable prognosis [27]), we also examined TINT changes in relation to molecular subtype. To start exploring the diagnostic potential of TINT changes, we analyzed the transcriptome of cancer-negative prostate biopsies obtained from men who received a high-grade cancer diagnosis within 2.5 years (cases where early diagnosis is particularly critical [28]), in comparison to cancer-containing biopsies, and biopsies from men who remained cancer-free for many years.

## Materials and methods

### Radical prostatectomy specimens (cohort 1)

Formalin fixed paraffin embedded (FFPE) tissue from 44 men who underwent radical prostatectomy between 2005 and 2019 was retrieved from the Department of Medical Biosciences, Pathology, Umeå University. Ethical approval was obtained from the local ethics committee (Dnr 03/482; 2013–57-31 M with amendments 2016–81-32 M, 2017–197-32 M), and all participants gave informed consent. Cases were selected to include a single tumor located in the peripheral zone on one side of the prostate, with the contralateral side confirmed to be cancer-free. Nineteen cases had extra-prostatic extension (pT3), of which 11 had positive surgical margins, and 25 cases were organ-confined (pT2). The ISUP grades were distributed as follows: 12 grade 1, 8 grade 2, 12 grade 3, 11 grade 4, and 1 grade 5. The men were aged 42–72 years (median 64) with a preoperative serum PSA ranging from 1.3–37 µg/l (median 8.3).

**Fig. 1:**
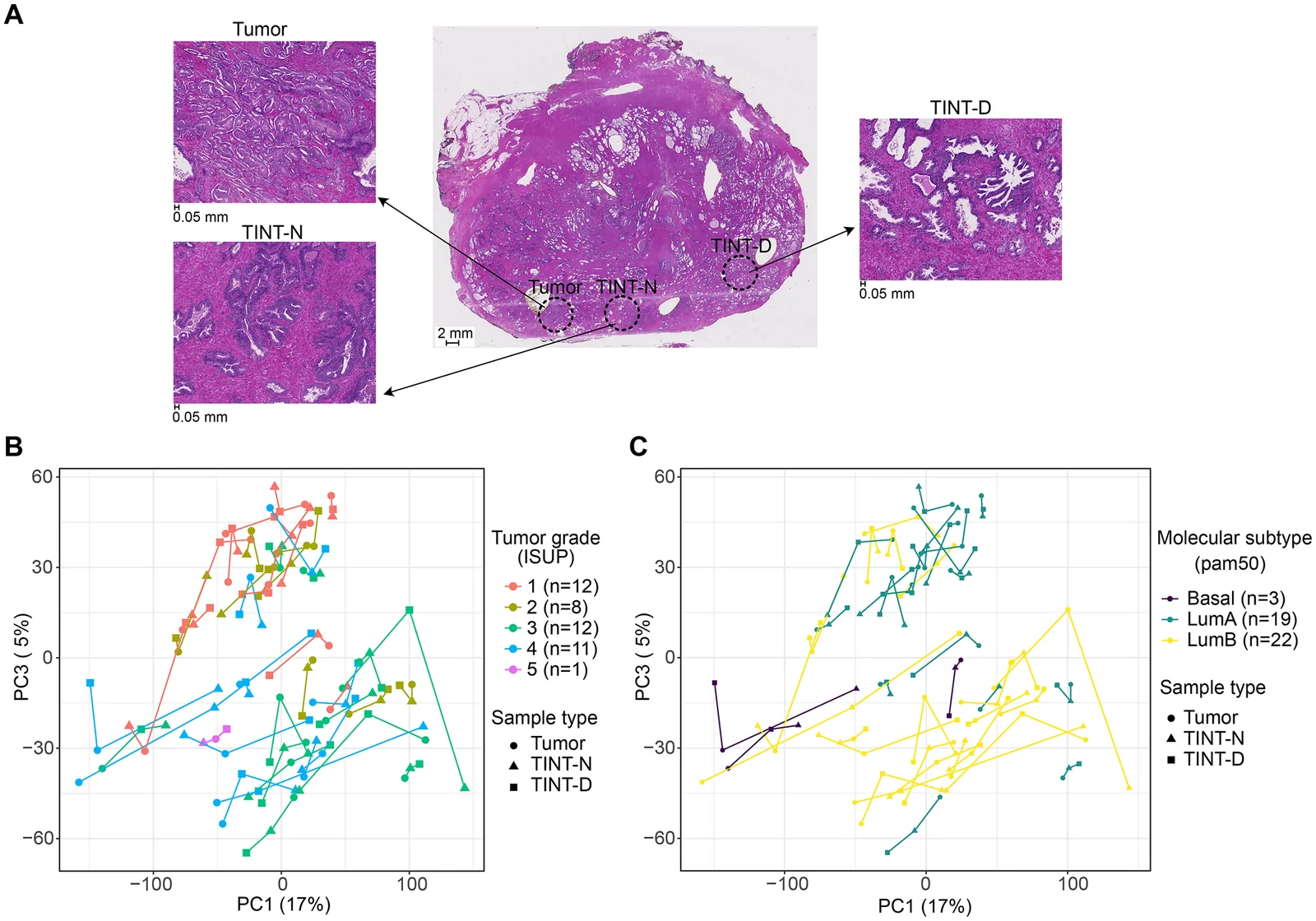
Principal component analysis (PCA) of transcriptomic profiles in radical prostatectomy samples. (**A**) Whole-mounted hematoxylin-eosin-stained section from radical prostatectomy specimen showing representative tissue areas of tumor tissue, histologically benign tissue from the peripheral zone *near* the tumor (TINT-N), and histologically benign tissue from the contralateral side *distant* to the tumor (TINT-D). PCA score plots based on mRNA profiles of protein coding-genes (*n* = 18886), colored according to ISUP grade (**B**) and molecular subtype (**C**). Lines connect samples (tumor, TINT-N and TINT-D) originating from the same patient (*n* = 44). The plots display the first and third principal components (the second component mainly captured an outlier)

Whole-mounted hematoxylin-eosin-stained prostate sections were examined to define three regions in each patient: (1) tumor tissue (*n* = 44), (2) histologically benign tissue from the peripheral zone *near* the tumor on the ipsilateral side (TINT-N, *n* = 44; 0.5–1 cm from the tumor), and (3) histologically benign tissue from the contralateral side more *distant* to the tumor (TINT-D, *n* = 42; >2 cm from the tumor) (Fig. 1A). Guided by these sections, a biopsy punch (Ø 6 mm) was used to encircle the selected Tumor, TINT-N and TINT-D tissue areas (~5 mm in diameter) on the corresponding paraffin-embedded prostate tissue blocks. Upon sectioning (10 µm, 5 sections/patient), the encircled areas were dissected into separate tubes for RNA extraction.

### Prostate biopsies (cohort 2)

A retrospective cohort of prostate biopsies (FFPE) was assembled by reviewing the pathology archives at Umeå University from 1998 to 2016. Ethical approval was obtained from the local ethical review board (Dnr 2010 366-31 M).

The study included men aged 52–84 years with elevated serum PSA levels (3.6–46 µg/l) whose initial systematic needle biopsies showed histologically benign prostate tissue (excluding cases with PIN or prostatitis). These men were selected based on two outcome groups: those later diagnosed with high-grade prostate cancer within 30 months (Bx-later-tumor, *n* = 46, ISUP 3 = 19, ISUP 4 = 19, and ISUP 5 = 8), and those who remained cancer-free for at least eight years despite repeat biopsies (Bx-control, *n* = 49). Bx-later-tumor and Bx-controls were matched for age, year of diagnosis, and initial PSA levels. 6–8 biopsies per patient were sectioned (10 µm, 1 section/biopsy) and pooled for RNA isolation. In addition, this dataset included tumor-containing biopsies from 28 patients (Bx-tumor, ISUP 2 = 1, ISUP 3 = 2, and ISUP 4 = 9, ISUP 5 = 16) with 1–4 biopsies/patient sectioned (10 µm, 1–4 sections/biopsy). All biopsies were obtained before MRI-guided biopsy became routine, as long follow-up time was needed. The study cohort is described in more detail in Chelebian et al. [29].

### RNA isolation, transcriptomic profiling, and data analysis

Total RNA was extracted using the Invitrogen PureLinkTM FFPE total RNA Isolation Kit (K156002, ThermoFisher) (cohort 1) or the Ionic FFPE to Pure RNA kit (cohort 2) on the Ionic purification system (Bionano Genomics), according to protocols [30]. RNA was quantified by a DeNovix DS 11 FXþSpectrophotometer (DeNovix Inc) and selected samples (TINT-N, *n* = 44) were quality assessed for DV200 using TapeStation (Agilent). RNA was amplified with the GeneChipTM WT Pico Reagent Kit and hybridized to Clariom D Human Transcriptome Arrays (Applied Biosystems, Thermofisher) at the Uppsala Array and Analysis Facility (Uppsala University, Uppsala, Sweden) (cohort 1) or the Bioinformatics and Expression Analysis core Facility (Karolinska Institute, Stockholm, Sweden) (cohort 2). Two separate array experiments were conducted, the first containing all the radical prostatectomy samples (T, TINT-N, TINT-D), and the second containing all the biopsies (Bx-later-tumor, Bx-control, Bx-tumor).

Raw data, for each experiment as well as both experiments together, was normalized using the Robust Multi-array Average (RMA) method [31] implemented in the R package oligo [32]. Principal component analysis (PCA) was performed using ropls [33] to give a visual overview of the data. Tumor molecular subtypes were determined with the PAM50 algorithm [27], classifying samples as LumA, LumB, or Basal (HER2 and normal subtypes were excluded). To minimize batch effects between datasets (radical prostatectomy and biopsy samples), normalization was performed with the removeBatchEffect function in the limma package [34], retaining information on both patient cancer status (Tumor/Bx-later-tumor/Bx-tumor vs. Bx-control) as well as sample status (non-malignant; TINT-N/TINT-D/Bx-later-tumor/Bx-control vs. tumor; Tumor/Bx-tumor).

Differential gene expression analyses were performed using limma [34]. Multiple samples from the same patient were handled using the duplicate-Correlation function. Three linear models were fitted to the radical prostatectomy dataset: (i) an **ISUP grade model** comparing sample ISUP status (a combination of ISUP group (1–2 vs. 3–5) and sample type (Tumor, TINT-N, or TINT-D), resulting in six categories (Tumor 1–2, TINT-N 1–2, TINT-D 1–2, Tumor 3–5, TINT-N 3–5, TINT-D 3–5) and adjusted for age and run (batch); (ii) a **PAM50 model** comparing sample PAM50 status (a combination of PAM50 classification (LumA, LumB, Basal) and sample type (Tumor, TINT-N or TINT-D)), adjusted for age and run; and (iii) a **PAM50 model adjusted for ISUP grade**: same as PAM50 model, but adjusted for age, run, and ISUP status. One model was fitted to the biopsy dataset: a **Bx model** comparing Bx-tumor, Bx-later-tumor, and Bx-control samples. Additional models combining radical prostatectomy and biopsy samples were also computed. These included (i) an **ISUP grade model with normal control tissue** comparing sample ISUP status (Tumor 1–2, TINT-N 1–2, TINT-D 1–2, Tumor 3–5, TINT-N 3–5, TINT-D 3–5, Bx-control) and adjusted for age, and (ii) a **PAM50 model with normal control tissue** comparing sample PAM50 status (Tumor LumA, TINT-N LumA, TINT-D LumA, Tumor LumB, TINT-N LumB, TINT-D LumB, Tumor Basal, TINT-N Basal, TINT-D Basal, Bx-control) and adjusted for age. Preprocessing and differential gene expression analyses were carried out in R version 4.4.2 (R Core Team, 202; https://www.R-project.org/)

Gene set enrichment analysis (GSEA) was performed using the hallmark gene sets in the Molecular Signature Database (MsigDB) via the msigdbr and fgsea R packages [35] as well as on GSEA software (v4.3.2, Broad Institute) [36], using a manually curated subset of MSigDB, C2: BIOCARTA gene sets, including only pathways related to adaptive immune cell function, cytokines and inflammation, immune checkpoints or co-stimulation, innate immune cells, and pattern recognition or inflammation signaling (*n* = 73) [37, 38]. Sorted *t* statistics of all protein-coding genes from differential gene expression analysis were used as pre-ranked input list. False discovery rate (FDR) q-value < 0.05 was used to select for significantly enriched pathways.

### Immunohistochemistry

Immunohistochemistry was performed on the automated Benchmark ULTRA platform (Ventana Medical systems) using the Universal DAB Detection kit and Hematoxylin as counterstain. Antigen retrieval was performed with Ultra Cell Conditioning 1 buffer.

Sections from radical prostatectomies were stained with Ki67 (marker for proliferation) (#790–4286 [9], ready-to-use, Ventana), CD8 (cytotoxic T-cells) (M7103, [C8/144B9], 1:50, DAKO), or CD68 (macrophages) (M0814, [KP1], 1:2000, DAKO).

The fraction of Ki67+ tumor and benign epithelial cells were measured as earlier described [39]. CD68+ and CD8+ cells per mm^2^ were quantified using the QuPath software version 0.4.3 [40]. Statistical analysis on was made with the Mann-Whitney U test and Spearman correlation (GraphPad Prism 10, and IBM SPSS Statistics 30).

## Results

### Global transcriptomic profiles of benign and tumor tissues in prostatectomy samples from men with unifocal prostate cancer

Transcriptomic profiles were obtained from 130 tissue samples derived from 44 radical prostatectomies. Tumor tissue, histologically benign prostate tissue 0.5–1.0 cm from the tumor (TINT-N; near), and histologically benign tissue > 2.0 cm from the tumor (TINT-D; distant) were analyzed (Fig. 1A). In two cases, TINT-D was unavailable. RNA quality from FFPE samples varied, with DV200 values ranging from 37.2% to 89.1% (TINT-N, *n* = 44, median 70.5%, IQR 62.5–75.2%), However, detection-above-background metrics showed no association with paraffin block storage time (Supplement Fig. 2), and overall array performance was comparable across the cohort (data not shown). Accordingly, all samples were included in downstream analyses.

After removal of control probe sets, 135,750 transcripts were analyzed, including 18,886 protein-coding genes. Principal component analysis (PCA) of transcriptomic profiles revealed two main trends: 1) Tumor and TINT samples from the same prostate mapped closely together, and 2) samples tended to separate by tumor grade and molecular subtype (Fig. 1B–C).

**Fig. 2:**
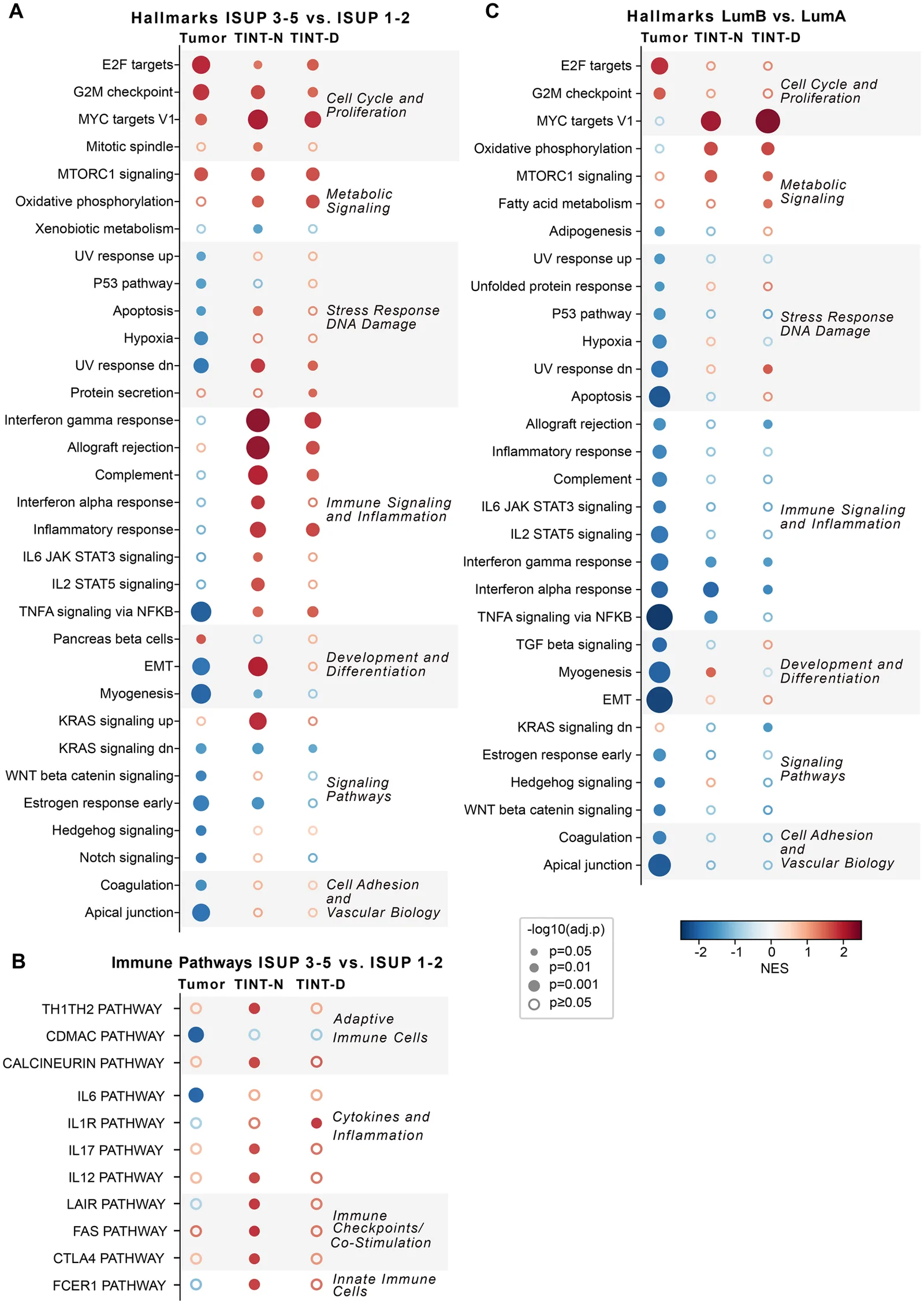
Gene set enrichment analysis (GSEA) of transcriptomic profiles in radical prostatectomy samples. GSEA was performed on mRNA profiles from radical prostatectomy tissues comparing (**i**) high-grade (ISUP 3–5, *n* = 24) vs. low-grade (ISUP1-2, *n* = 20) samples within each tissue compartment (tumor, TINT-N, and TINT-D) (hallmark pathways in (**A**) and immune-related biocarta pathways in (**B**), and (ii) molecular subtypes LumB (*n* = 22) vs. LumA (*n* = 19) (**C**). The color scale indicates significantly enriched (red) or suppressed (blue) hallmarks, based on normalized enrichment scores (adjusted *p* < 0.05)

To identify functional differences potentially related to the trend observed for tumor grade (Fig. 1B), we performed GSEA on tumor and adjacent benign (TINT-N, and TINT-D) regions, comparing high-grade (ISUP 3–5) and low-grade (ISUP 1–2) tumors. High-grade tumors were enriched for proliferation-related hallmarks (*E2F targets, G2M checkpoint, MYC targets V1*) and metabolic *MTORC1 signaling*, with similar enrichments seen in TINT-N and TINT-D (Fig. 2A). Many other hallmark gene sets were suppressed in high- vs low-grade tumors (Fig. 2A). Notably, all inflammation-related gene sets were enriched in TINT regions, some restricted to TINT-N (Fig. 2A), suggesting a tumor proximity-dependent immune response in high-grade tumors. More in-depth analysis based on immune-related Biocarta pathways confirmed upregulation of inflammatory and regulatory pathways in TINT-N, consistent with a state of chronic inflammation (Fig. 2B).

To explore whether T cell and macrophage activation-associated gene expression differed according to tumor grade, expression of selected cytotoxic and immune regulatory genes was normalized to the T-cell marker CD3 and the macrophage marker CD68. The resulting ratios were compared between high-grade and low-grade cases separately within tumor, TINT-N and TINT-D tissue areas (Supplement table 1). Compared with TINT-N in low-grade cases, TINT-N adjacent to high-grade tumors exhibited significantly lower expression of immune-regulatory genes (CTLA4, PDCD1) and cytotoxic genes (IFNG, GNLY, GZMA, GZMB, PRF1) following CD3-normalization, as well as lower expression of several myeloid-associated activation markers (CD274, MRC1, IDO1, CXCL10) following CD68-normalization. Most differences were restricted to TINT-N, with minimal changes in more distal tissue (TINT-D) and some alterations within the tumor compartment (Supplement Table 1). These findings suggest that, although TINT-N in high-grade tumors show higher overall immune-related gene expression than TINT-N in low-grade cases (Fig. 2A–B), the relative immune cell activity could be reduced in the vicinity of high-grade tumors.

Paired analyses showed that the transcriptomic distinction between TINT-N and tumor was more pronounced in ISUP 3–5 than in ISUP 1–2 cases (29 vs. 14 significant hallmark gene sets), including several immune- and inflammation-related sets that reached significance only in high-grade cases (Supplement Tables 2, 3). Direct comparison of TINT-D with TINT-N showed the same grade-dependence: six hallmarks, including epithelial-mesenchymal transition and several immune-related sets, differed by distance in ISUP 3–5 cases versus only one (myogenesis) in ISUP 1–2 cases (Supplement Table 4). Consistent with this, proliferation-, metabolism- and immune-related hallmarks were all lower in TINT-N from low-grade tumors than in TINT-D from high-grade tumors (Supplement Table 5).

We next examined the influence of tumor molecular subtypes (19 LumA, 22 LumB, 3 Basal) on TINT transcriptomes. LumA tumors were predominantly low-grade (12 ISUP 1–2, 7 ISUP 3–5), while LumB (7 ISUP 1–2, 15 ISUP 3–5) and Basal (1 ISUP 1–2, 2 ISUP 3–5) were mainly high-grade. GSEA was performed to compare LumB and LumA cases, while basal cases were excluded due to low number. LumB tumors were enriched for proliferation hallmarks (*E2F targets, G2M checkpoint*), whereas stress response, inflammation, differentiation, and signaling pathways were generally suppressed (Fig. 2C). In LumB-associated TINT, some proliferation- (*MYC targets V1*) and metabolism- (*oxidative phosphorylation, MTORC1 signaling, fatty acid metabolism*) related hallmarks were enriched, while some inflammation-related hallmarks (*interferon gamma response, interferon alpha response, TNFA signaling*) were suppressed (Fig. 2C). Thus, pathways enriched in ISUP 3–5 TINT-N compared to ISUP 1–2 TINT-N but suppressed in LumB TINT-N compared to LumA TINT-N were inflammation-related, highlighting tumor-grade- and subtype-specific alterations of the benign prostate tissue (Fig. 2A–C). As LumB tumors had a higher ISUP grade than LumA tumors (*p* = 0.045, Chi-square test), we repeated the differential expression and GSEA analyses with grade adjustment, which yielded similar trends (Supplement Fig. 2).

### Immunohistochemical analysis of proliferation and immune-cell infiltration in TINT

As increased cell proliferation and activated immune responses emerged as prominent features of TINT, we used immunohistochemistry to quantify the fraction of Ki67+ epithelial cells and the densities of CD68+ macrophages and CD8+ T-cells in tumor tissue and TINT. The fraction of Ki67+ epithelial cells was higher in tumor, TINT-N and TINT-D samples from high-grade compared to low-grade cases (Supplement Fig. 3). Moreover, Ki67 labeling in tumors correlated with that in both TINT-N (Rs = 0.767, *p* < 0.001) and in TINT-D (Rs = 0.759, *p* < 0.001). The density of CD68+ macrophages was higher in tumor and TINT-N tissues in high-grade compared with low-grade cases (Supplement Fig. 3). In addition, CD68+ cell density in tumors was correlated to that in TINT-N (Rs = 0.542, *p* < 0.001) and TINT-D (Rs = 0.448, *p* = 0.003). Notably, in TINT some CD68+ cells invaded into the glandular epithelium. The density of CD8+ T-cells was significantly higher in TINT-N and TINT-D from high-grade compared with low-grade cases, whereas no significant differences were observed in tumor tissue (Supplement Fig. 3). Furthermore, CD8+ cell density in tumors was correlated to that in TINT-N (Rs = 0.344, *p* = 0.022) and TINT-D (Rs = 0.476, *p* = 0.001).

The fraction of Ki67+ epithelial cells was higher in tumor, TINT-N and TINT-D in LumB compared with LumA cases, whereas the densities of CD68+ and CD8+ cells were similar in LumB and LumA cases, except for CD68 that was significantly higher in TINT-D of LumA compared to LumB (Supplement Fig. 4).

### The transcriptome of cancer-negative biopsies reflects subsequent diagnosis of high-grade cancer

To explore whether early transcriptomic alterations in histologically benign tissue are indicative of future cancer diagnosis, we analyzed transcriptomic profiles from biopsy samples of men who were diagnosed with high-grade cancer within 2.5 years (Bx-later-tumor; left and right side biopsies combined), biopsies containing cancer at the time of sampling (Bx-tumor), and biopsies from men who remained cancer-free (Bx-control).

**Fig. 3:**
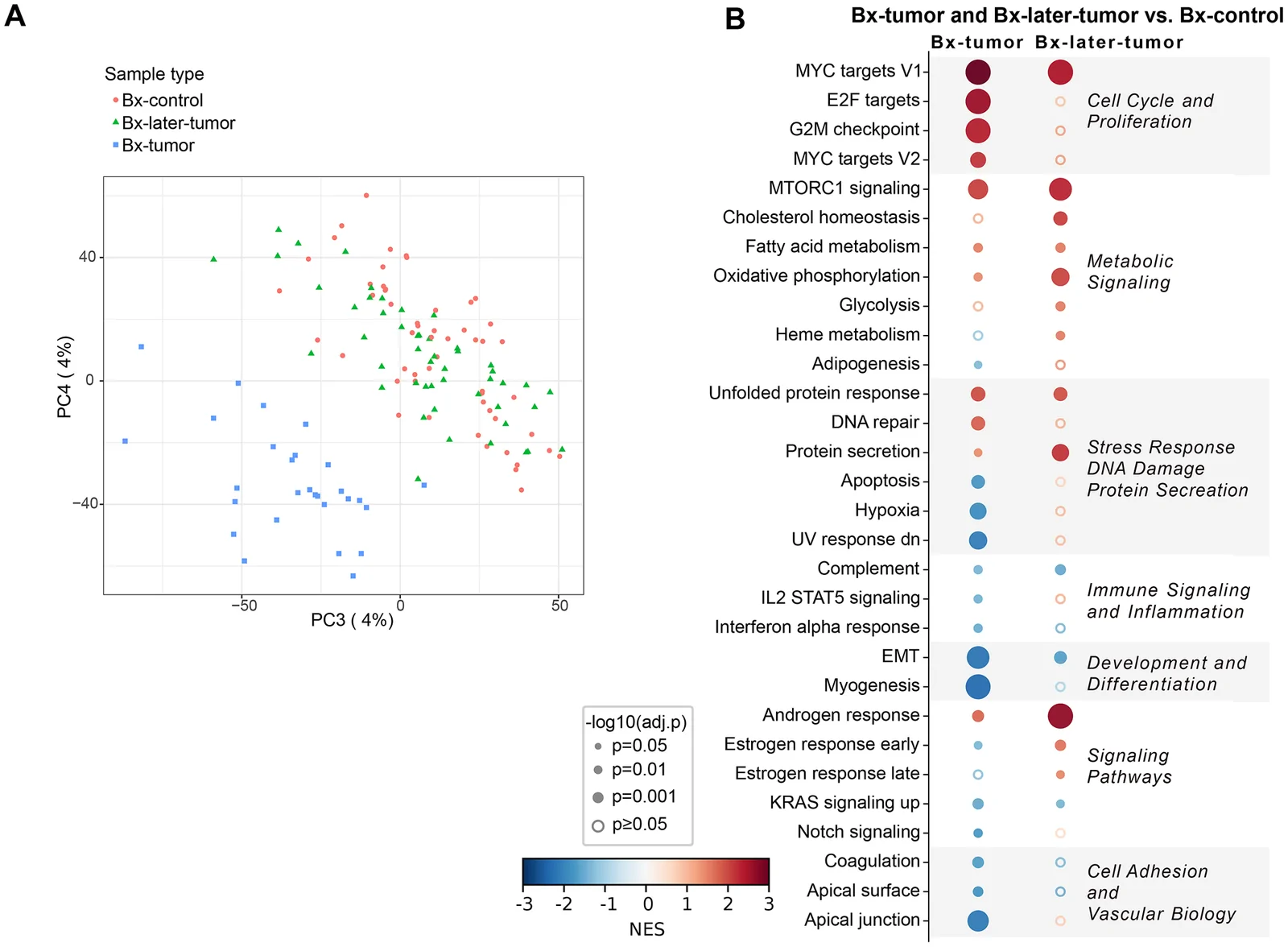
Principal component analysis (PCA) and gene set enrichment analysis (GSEA) of transcriptomic profiles in biopsy samples (**A**) PCA score plot based on mRNA profiles of protein-coding genes in biopsy samples from men who were diagnosed with high-grade cancer within 2.5 years (bx-later-tumor, *n* = 46), biopsies containing cancer at the time of sampling (bx-tumor, *n* = 28), and biopsies from men who remained cancer-free for at least 8 years (bx-control, *n* = 49). (**B**) GSEA of mRNA profiles in transcriptomic Bx-tumor and Bx-later-tumor vs Bx-controls. The color scale reflects significantly induced (red) and suppressed (blue) hallmarks, according to normalized enrichment scores (adjusted *p* < 0.05)

As expected, Bx-tumor samples were clearly separated from the benign tissue samples (Bx-later-tumor and Bx-control) by PCA (Fig. 3A), and GSEA revealed that Bx-tumor samples were enriched for hallmarks related to proliferation (*MYC targets V1 & V2, E2F targets, G2M checkpoint*) and metabolism (*MTORC1 signaling, fatty acid metabolism, oxidative phosphorylation*), while hallmarks related to immune response, development and differentiation, cell adhesion and vascular biology were generally decreased compared to Bx-controls (Fig. 3B).

However, Bx-later-tumor samples did not separate from Bx-controls, indicating limited transcriptomic differences between these benign groups (Fig. 3A). Nevertheless, Bx-later-tumor samples showed a general enrichment of metabolic hallmarks compared to Bx-controls (Fig. 3B), suggesting that metabolic alterations may arise in histologically benign tissue before tumor development, or because of signaling from an undetected tumor elsewhere in the prostate.

In addition, Bx-later-tumor samples shared other hallmark changes with Bx-tumor, including enrichment of *Myc targets V1, Unfolded protein response, Protein secretion and Androgen response,* along with decreased *Complement, EMT,* and *KRAS signaling* (Fig. 3B). Unlike the TINT signatures observed in established high-grade tumors (Fig. 2A–B), the Bx-later-tumor samples did not exhibit increased immune signaling or inflammation-related hallmarks (Fig. 3B), suggesting that such responses emerge later in tumor progression.

### Comparing the tumor and TINT transcriptomes in radical prostatectomy specimens to that in biopsies

We then compared transcriptomes of tumor and adjacent benign (TINT-N, TINT-D) regions from radical prostatectomies with those of Bx-control biopsies (after data normalization allowing comparison between the two cohorts).

**Fig. 4:**
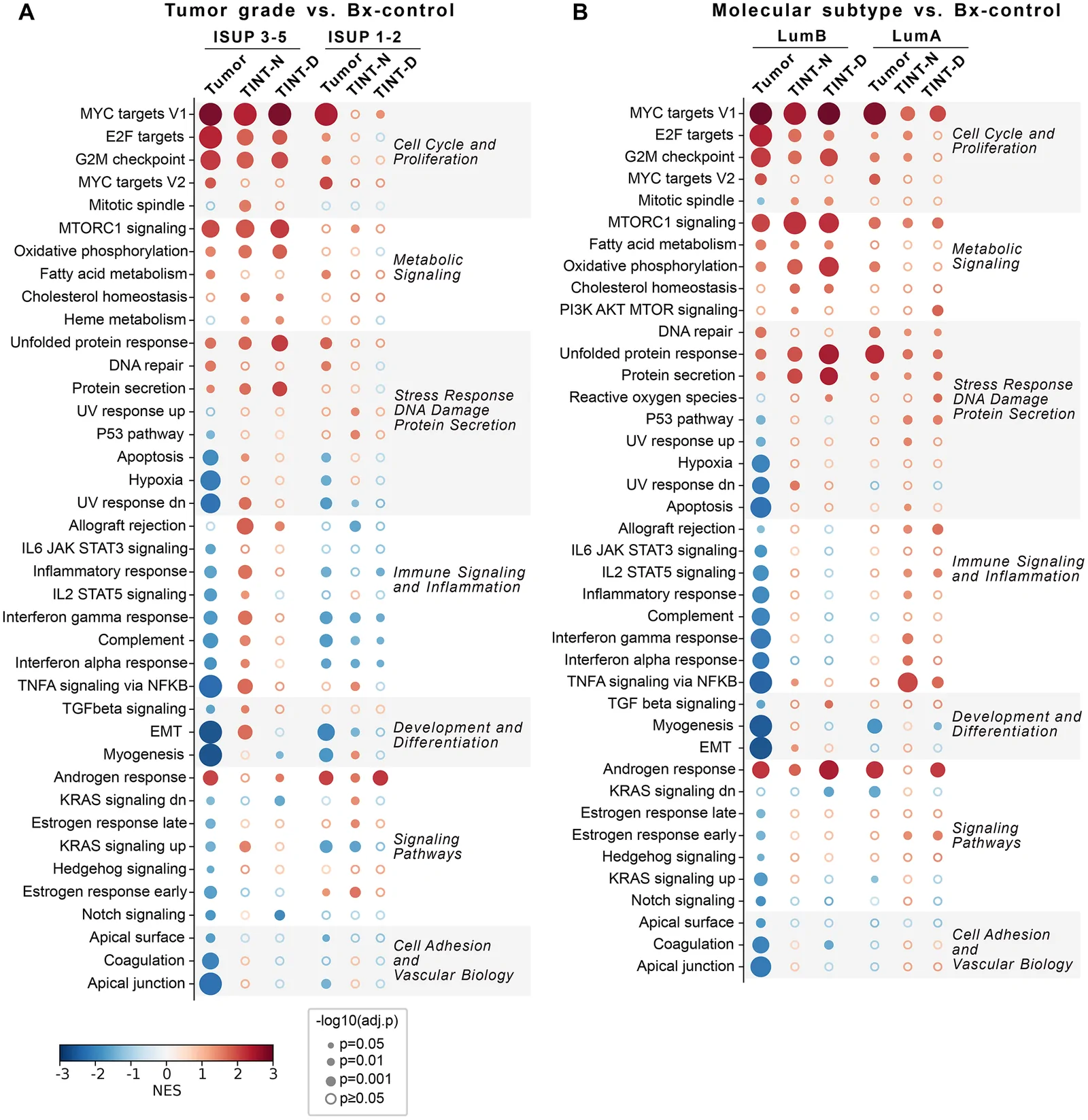
Gene set enrichment analysis (GSEA) of transcriptomic profiles in tumor and TINT samples of radical prostatectomies compared to control biopsies. Enriched hallmarks in tumor, TINT-N and TINT-D samples of radical prostatectomies according to (**A**) tumor grade; ISUP 3–5 (*n* = 24) and ISUP 1–2 (*n* = 20) vs control biopsies (bx-control, *n* = 49), and (**B**) molecular subtype; LumB (*n* = 22) and LumA (*n* = 19) vs control biopsies (bx-control, *n* = 49). The color scale reflects significantly induced (red) and suppressed (blue) hallmarks, according to normalized enrichment scores (adjusted *p* < 0.05)

High-grade tumors in radical prostatectomies showed similar but not identical hallmark changes as Bx-tumors in comparison to Bx-controls (Figs. 4A and 3B). Consistent with findings in Fig. 3B, gene sets related to proliferation (*MYC targets V1, E2F targets, G2M checkpoint*) and metabolism (*MTORC1 signaling, oxidative phosphorylation*) were enriched in high-grade tumors and their adjacent TINT-N and TINT-D areas compared with Bx-controls, whereas these changes were less pronounced in TINT regions of low-grade tumors (Fig. 4A). Also, many inflammation-related pathways were suppressed in both high- and low-grade tumors vs. Bx-controls (Fig. 4A) in line with findings in Bx-tumor tissue (Fig. 3B). However, high-grade TINT-N showed strong inflammatory enrichment compared with TINT-D, while low-grade TINT displayed weak or suppressed inflammatory activation (Fig. 4A), again supporting a tumor proximity-dependent immune response in high-grade tumors. ISUP 3–5-associated TINT-N also showed enrichment of *Unfolded protein response*, *Protein secretion*, *Apoptosis*, *UV response*, *TGFβ* signaling, *EMT*, and *KRAS signaling* (Fig. 4A).

Comparing tumor and TINT areas by molecular subtype, both LumA and LumB tumors showed enrichment of proliferation and metabolic pathways compared to Bx-controls, while inflammation was exclusively suppressed in LumB tumors. LumA TINT-N, in contrast to LumB TINT-N, showed enrichment of inflammatory gene sets (Fig. 4B).

## Discussion

This study shows that gene expression in benign regions of tumor-bearing prostates varies with tumor proximity, grade, and molecular subtype, and that these changes occur in pathway-specific patterns. Essentially, benign tissue from prostates with cancer differs from prostate tissue in tumor-free men as earlier reported using various types of prostate tissue as “normal” controls [1, 4, 18], and high-grade or LumB tumors are associated with more extensive alterations than less aggressive tumors. Although our Bx-control group does not represent a truly healthy reference population, as all these men had elevated PSA levels prompting biopsy, these men remained free from clinically detectable prostate cancer during long-term follow-up and therefore provide a clinically relevant cancer-free comparator. Thus, benign samples adjacent to ISUP grade 3–5 or LumB tumors likely differs biologically from true “normal” prostate tissue and TINT associated with lower-grade tumors.

These findings are largely consistent with our experimental rat studies demonstrating that tumors with different aggressiveness induce distinct proximity-dependent changes in surrounding prostate tissue resembling those now observed in patients [2, 6–9]. Prostate tumors, particularly high-metastatic cases, apparently release factors, in part delivered in exosomes, that affect both the epithelium and stroma in the entire organ [1, 8, 9, 41, 42]. Some of these factors may also contribute to pre-metastatic niche formation in distant organs [7, 43, 44]. Transcriptomic changes observed in non-malignant regions of a tumor-bearing human prostate, here and in [1], are therefore consistent with tumor-induced signaling (TINT) although pre-existing cancer-field effects cannot be excluded. Still, copy-number alterations in benign tissue close to an ISUP grade 4 tumor [45] indicate that both mechanisms may operate, although tumor-induced genetic changes in benign tissues (a TINT effect) also remain possible [46, 47].

We identified two main categories of TINT-related changes. The first involves parallel enrichment in the tumor and adjacent benign tissue, as were seen for hallmark gene sets related to increased proliferation and altered metabolism. In line with this cell proliferation in tumor and TINT epithelial cells was more increased in high-grade than in low-grade cases. A similar pattern, characterized by increased epithelial cell proliferation, vascular growth, and altered metabolism, was also seen in our rat TINT model [2, 6, 7, 11, 42]. This may indicate that tumor-secreted factors promoting proliferation and metabolic activity within tumors also influence surrounding benign tissues potentially preparing them for subsequent tumor invasion [2], but the exact cellular targets remain to be defined. Metabolic alterations in TINT are known to vary with tumor grade and proximity to the tumor [14, 23, 48–50]. Normal prostate epithelial cells uniquely secrete citrate, but tinted tumor-adjacent non-malignant prostate epithelial cells instead, like tumor cells, metabolize citrate to enhance energy production and metabolism [23, 51]. Factors altered in both tumor and TINT in parallel may represent potential targets for intervention, and if secreted they may represent candidate biomarkers in circulation, given that the TINT volume is substantially larger than the tumor itself [2, 10].

The second category of transcriptomic TINT changes, primarily involving immune signaling and inflammation, seem specifically enriched or altered in TINT regions, particularly those adjacent to high-grade tumors, while largely absent or suppressed within tumor areas, as previously reported [19, 23, 52–57]. Compared with TINT from low-grade cases, TINT surrounding high-grade prostate tumors was characterized by increased CD8+ T-cell and CD68+ cell infiltration together with lower expression of several cytotoxic, immune regulatory, and myeloid activation markers following normalization to CD3 or CD68, respectively. Together with the GSEA results, these observations suggest that aggressive tumors are associated with altered immune signaling in adjacent benign tissue. Benign tissue in the vicinity of high-grade tumors seemed to attract immune cells to a higher level than benign tissue adjacent to low-grade tumors, but immune cell activities in high grade areas appeared reduced, indicating an immune suppressed microenvironment. However, because the current observations are associative, based on bulk transcriptomic data from patient material, we can neither establish whether these changes reflect altered immune-cell function nor define their role in tumor progression. Interestingly, benign epithelial cells adjacent to high-grade cancer specifically, enriched for the club-like epithelial cell type, demonstrate increased secretion of pro-inflammatory chemokines [19, 23]. Macrophage-depletion experiments in our rat TINT-model suggest that the CD68+ macrophages accumulate in TINT, and in contrast to those within the tumor, promote vascular growth in TINT and thus tumor growth [11, 46, 58]. A similar role for CD68+ macrophages in humans is not unlikely. As some CD68+ cells apparently migrate into benign glands they could be involved in reshaping this tissue compartment prior to tumor invasion.

These results are in accordance with our earlier studies in rats showing both tumor-mirroring changes and TINT-specific immune activation [7, 9]. In the rat TINT model, tumors with high metastatic potential induced more pronounced macrophage and neutrophil infiltration, and transcriptomic changes consistent with an inflammatory response in TINT, than less aggressive tumors [6, 11]. How the immune activation in high-grade associated TINT, together with changes in metabolism and cell proliferation, ultimately influences and possibly stimulates tumor growth remains to be clarified but our findings may suggest that potentially useful immunomodulatory targets primarily reside in tumor-adjacent and not intra-tumoral tissue.

Differences in TINT patterns were also observed between LumA and LumB cases, where LumA, but not LumB, showed enrichment of immune-related pathways. Histological grade and molecular subtype thus appear to influence TINT in partly distinct ways, possibly reflecting different biological properties, with grade possibly relating to differentiation and growth pattern and subtype to intrinsic molecular and cellular features. Whether a stronger immune response surrounding LumA tumors contributes to their more favorable prognosis remains to be determined.

Few prior studies have assessed gene expression in tumor-adjacent prostate tissue in relation to grade or prognosis, and none to tumor multifocality. Magi-Galluzzi et al. observed androgen signaling and stress responses in benign tissue > 3 mm from mainly ISUP 1 tumors in men with PSA relapse [24]. Zhou et al. reported relapse-associated activation in benign tissue (distance unspecified) of myogenesis, EMT, angiogenesis, KRAS signaling, and oxidative phosphorylation, while low-risk cases showed increased immune activity [25]. Limited overlap with our findings in high-grade cases may reflect variation in tumor multifocality, sampling distance, and tissue handling (see below).

Several authors have proposed using transcriptomic, metabolic, or morphological TINT changes to guide re-biopsy decisions for men with elevated PSA but negative biopsies [2, 3, 59, 60]. Methylation-based assays such as ConfirmMDX (https://www.mdxhealth.com) and own AI-based histological analyses of benign standard hematoxylin-eosin-stained biopsies from the same cohort as in the present study [61] support this concept. Although MRI-guided biopsy has reduced overt sampling errors [62], our current findings suggest that detection of TINT changes, that apparently involve epithelial cells, stroma cells and the extracellular matrix as earlier reported [11–13, 15, 17, 19, 61], could further support identification of aggressive cancers. The best, most cost-effective, and clinically applicable approach for detecting such TINT changes remains to be determined. However, one plausible strategy may be the use of histological AI applied to standard prostate biopsy sections [61], rather than more costly and technically complicated transcriptomic analyzes.

We observed both transcriptomic similarities and differences between TINT areas of prostatectomy tissue and benign biopsy samples from men later diagnosed with high-grade tumors. Similarities were related to metabolic responses while signs of increased proliferation and immune responses were absent in the biopsies, which might indicate a metabolic switch that presides proliferative and immunological effects in TINT tissue. Alternatively, differences may reflect tumor size at biopsy, distance to tumor, challenges inherent to comparing biopsy with prostatectomy tissue (see below), or basically that no tumor was present at the time for initial biopsy and that observed changes instead may represent cancer field effects.

Tumor and TINT samples from the same patient clustered together in unsupervised analysis based on transcriptomic similarities. This could reflect a combination of TINT and cancer-field effects, although other factors probably also play a role. Previous work shows that differences in patient characteristics and pre-analytic tissue handling—such as those between biopsy and prostatectomy samples from the same individual—can generate greater transcriptomic variation than the differences observed between tumor and benign tissue [63]. This limits the potential of detecting minor differences between patients, and it also complicates the use of “normal” prostate tissue as a reference, especially when common comparator sources (autopsy tissue, donor tissue, prostate tissue obtained from bladder-cancer patients) differ substantially in pre-analytic handling and clinical context. The main conclusions drawn in the current study, that TINT changes are related to tumor grade, molecular subtype and proximity, are however not dependent on comparisons of tissue sampled by needle biopsy and after prostatectomy.

Understanding what drives TINT-changes remains crucial. Our rat studies suggest potential upstream regulators including CXCL12, TNFα, TGFβ1, IFNγ, IL1β, S100A8/9, prolactin, and hypoxia [7]. Similar factors have been implicated in human samples by Aran et al. [1]. Many of these factors are linked to immune regulation, inflammation, macrophages, tissue reconstruction and repair, suggesting them also as central drivers of TINT. Responses that are particularly prominent around aggressive tumors [2]. Further studies are needed to determine how TINT changes are induced, their biological significance, if they can be mimicked by other prostate pathologies, and whether they can be therapeutically targeted. Analyses also at the protein-level are probably necessary, given the modest correlation between mRNA and protein levels in prostate tissue [64], as well as experimental studies to define the causal mechanisms involved. As immune responses seem important, experimental models using immune-competent animals are probably needed.

**In conclusion,** this study demonstrates that histologically benign tissue in human tumor-bearing prostates exhibits distinct transcriptomic alterations associated with tumor proximity, grade, and molecular subtype. Alterations in proliferation-, metabolism-, and immune-related pathways differed according to tumor characteristics, supporting the concept that biologically distinct TINT patterns are associated with different tumor phenotypes. While our experimental rat studies show that prostate tumors can induce similar alterations in non-malignant prostate tissue, the present analyses of patient material are observational and therefore establish tumor-associated rather than causal relationships. Further studies are needed to define the mechanisms underlying these associations, assess how TINT manifests in multifocal disease, and evaluate whether TINT-related alterations could aid in the early detection of aggressive tumors while providing a basis for further exploration of tumor-host interactions and potential targets for intervention.

## Electronic supplementary material

Below is the link to the electronic supplementary material.

Supplementary material 1

Supplementary material 2

## Data Availability

The transcriptomic data generated and analyzed during this study have been deposited in Array Express accession E-MTAB-17721 at https://www.ebi.ac.uk/biostudies/arrayexpress. The data has been fully anonymized and is provided with necessary, unidentifiable sample metadata. The code used in this study is openly available in the GitHub repository at https://github.com/NBISweden/TINT2026.

## Abbreviation

TINT: Tumor-instructed normal tissue
Bx: Biopsy, LumA; Luminal A
LumA: Luminal A
LumB: Luminal B
PCA: Principal component analysis
GSEA;: Gene set enrichment analysis
