# Supplementary material for: Gene expression profiles in benign prostate tissue reflect tumor proximity, grade, and molecular subtype
Source: J Transl Med. 2026 Oct 3;24:1252. doi: 10.1186/s12967-026-09035-8 (PMC13640278; doi:10.1186/s12967-026-09035-8)
Supplement: Supplementary file 1 [file 12967_2026_9035_MOESM1_ESM.pdf]

Supplement Table 1: Associations between gene expression of selected immune-related genes (normalized to CD3 [T cells] and CD68 [macrophages]) and tumor grade (ISUP 3–5 vs. 1–2). Data are presented as log2 fold change with 95% confidence intervals and corresponding p-values. Age and sequencing run were included as covariates in the model.

Ratio	**3-5 vs 1-2, Tumor**		**3-5 vs 1-2, TINT-N**		**3-5 vs 1-2, TINT-D**	
	log2FC	*p*	log2FC	*p*	log2FC	*p*
CTLA4_CD3	-0.37 (-0.71, -0.03)	0.010	-0.41 (-0.75, -0.06)	0.005	-0.26 (-0.60, 0.09)	0.082
PDCD1_CD3	-0.40 (-0.81, 0.02)	0.027	-0.54 (-0.95, -0.12)	0.003	-0.29 (-0.70, 0.13)	0.113
FAS_CD3	-0.26 (-0.64, 0.13)	0.117	-0.16 (-0.54, 0.22)	0.327	-0.21 (-0.60, 0.17)	0.195
IFNG_CD3	-0.06 (-0.47, 0.34)	0.706	-0.35 (-0.75, 0.06)	0.043	-0.17 (-0.58, 0.23)	0.319
GNLY_CD3	-0.28 (-0.68, 0.12)	0.095	-0.45 (-0.85, -0.05)	0.008	-0.42 (-0.82, -0.01)	0.015
GZMA_CD3	0.05 (-0.26, 0.35)	0.706	-0.27 (-0.57, 0.04)	0.037	-0.03 (-0.34, 0.28)	0.807
GZMB_CD3	-0.33 (-0.71, 0.06)	0.047	-0.42 (-0.81, -0.03)	0.011	-0.22 (-0.62, 0.17)	0.180
PRF1_CD3	-0.40 (-0.79, -0.02)	0.014	-0.46 (-0.84, -0.07)	0.005	-0.28 (-0.67, 0.11)	0.096
S100A9_CD68	-0.15 (-0.53, 0.22)	0.335	-0.31 (-0.69, 0.07)	0.050	0.02 (-0.36, 0.41)	0.896
CD274_CD68	-0.03 (-0.39, 0.34)	0.863	-0.49 (-0.85, -0.12)	0.001	-0.09 (-0.46, 0.28)	0.559
SPP1_CD68	0.22 (-0.50, 0.93)	0.475	0.25 (-0.46, 0.97)	0.400	0.01 (-0.71, 0.73)	0.974
MRC1_CD68	-0.01 (-0.32, 0.30)	0.943	-0.36 (-0.67, -0.05)	0.006	0.04 (-0.27, 0.36)	0.747
CD163_CD68	-0.38 (-1.29, 0.54)	0.350	0.38 (-0.54, 1.30)	0.343	-0.07 (-0.99, 0.86)	0.870
IDO1_CD68	-0.15 (-0.51, 0.21)	0.323	-0.35 (-0.71, 0.01)	0.020	-0.14 (-0.50, 0.23)	0.373
CXCL10_CD68	-0.24 (-0.59, 0.12)	0.111	-0.33 (-0.68, 0.03)	0.027	-0.08 (-0.44, 0.28)	0.592

Log2 ratios were calculated from RMA-normalized expression data as differences in log2 expression values. CD3-normalized ratios were calculated using the mean expression of CD3D and CD3E. Associations with ISUP grade (3-5 vs 1-2) were assessed using linear mixed-effects models with patient as a random intercept.

Supplement Table 2. Hallmarks in TINT-N vs. Tumor in cases with ISUP 1-2			
**Pathway**	**P value**	**Adj. P value**	**NES**
HALLMARK_MYOGENESIS	2.10E-13	5.25E-12	2.33
HALLMARK_CHOLESTEROL_HOMEOSTASIS	1.05E-05	1.76E-04	2.05
HALLMARK_KRAS_SIGNALING_DN	2.30E-04	2.87E-03	1.66
HALLMARK_HYPOXIA	4.33E-04	4.33E-03	1.62
HALLMARK_TNFA_SIGNALING_VIA_NFKB	5.39E-04	4.49E-03	1.60
HALLMARK_EPITHELIAL_MESENCHYMAL_TRANSITION	6.84E-04	4.89E-03	1.59
HALLMARK_APICAL_JUNCTION	1.31E-03	7.30E-03	1.57
HALLMARK_APOPTOSIS	3.50E-03	1.46E-02	1.54
HALLMARK_P53_PATHWAY	1.28E-02	4.58E-02	1.42
HALLMARK_DNA_REPAIR	3.35E-03	1.46E-02	-1.51
HALLMARK_UNFOLDED_PROTEIN_RESPONSE	4.71E-03	1.81E-02	-1.54
HALLMARK_ANDROGEN_RESPONSE	1.07E-03	6.71E-03	-1.65
HALLMARK_MYC_TARGETS_V2	3.11E-03	1.46E-02	-1.68
HALLMARK_MYC_TARGETS_V1	1.02E-15	5.11E-14	-2.48

Supplement Table 3. Hallmarks in TINT-N vs. Tumor in cases with ISUP 3-5			
**Pathway**	**P value**	**Adj. P value**	**NES**
HALLMARK_EPITHELIAL_MESENCHYMAL_TRANSITION	3.80E-27	1.90E-25	2.69
HALLMARK_TNFA_SIGNALING_VIA_NFKB	5.76E-22	1.44E-20	2.54
HALLMARK_UV_RESPONSE_DN	7.49E-14	8.51E-13	2.39
HALLMARK_INTERFERON_GAMMA_RESPONSE	1.06E-14	1.76E-13	2.28
HALLMARK_HYPOXIA	8.51E-14	8.51E-13	2.23
HALLMARK_MYOGENESIS	1.79E-13	1.50E-12	2.22
HALLMARK_APOPTOSIS	2.05E-10	1.02E-09	2.18
HALLMARK_INFLAMMATORY_RESPONSE	2.14E-12	1.53E-11	2.17
HALLMARK_COMPLEMENT	2.45E-12	1.53E-11	2.16
HALLMARK_INTERFERON_ALPHA_RESPONSE	5.06E-08	2.30E-07	2.15
HALLMARK_APICAL_JUNCTION	2.01E-11	1.12E-10	2.11
HALLMARK_COAGULATION	7.93E-08	3.30E-07	2.02
HALLMARK_TGF_BETA_SIGNALING	7.54E-05	2.35E-04	1.95
HALLMARK_IL2_STAT5_SIGNALING	1.45E-07	5.57E-07	1.89
HALLMARK_IL6_JAK_STAT3_SIGNALING	7.99E-05	2.35E-04	1.86
HALLMARK_KRAS_SIGNALING_UP	9.22E-07	3.29E-06	1.84
HALLMARK_HEDGEHOG_SIGNALING	5.48E-03	1.10E-02	1.66
HALLMARK_MITOTIC_SPINDLE	2.97E-04	7.83E-04	1.62
HALLMARK_ALLOGRAFT_REJECTION	4.21E-04	1.05E-03	1.61
HALLMARK_P53_PATHWAY	1.96E-03	4.52E-03	1.51
HALLMARK_WNT_BETA_CATENIN_SIGNALING	1.97E-02	3.28E-02	1.49
HALLMARK_ESTROGEN_RESPONSE_EARLY	4.09E-03	8.52E-03	1.48
HALLMARK_CHOLESTEROL_HOMEOSTASIS	1.94E-02	3.28E-02	1.47
HALLMARK_HEME_METABOLISM	6.75E-03	1.30E-02	1.45
HALLMARK_UV_RESPONSE_UP	9.84E-03	1.76E-02	1.45
HALLMARK_ESTROGEN_RESPONSE_LATE	2.06E-02	3.32E-02	1.38
HALLMARK_E2F_TARGETS	3.58E-03	7.79E-03	-1.42
HALLMARK_G2M_CHECKPOINT	1.99E-03	4.52E-03	-1.44
HALLMARK_MYC_TARGETS_V2	8.75E-03	1.62E-02	-1.53
HALLMARK_MYC_TARGETS_V1	2.62E-04	7.28E-04	-1.56
HALLMARK_ANDROGEN_RESPONSE	8.11E-06	2.70E-05	-1.94

Supplement Table 4. Hallmarks in TINT-D vs. TINT-N			
ISUP 1-2			
**Pathway**	**P value**	**Adj. P value**	**NES**
HALLMARK_MYOGENESIS	6.94E-04	3.40E-02	-1.42
ISUP 3-5			
**Pathway**	**P value**	**Adj. P value**	**NES**
HALLMARK_EPITHELIAL_MESENCHYMAL_TRANSITION	1.79E-11	8.95E-10	-2.00
HALLMARK_KRAS_SIGNALING_UP	1.10E-04	2.76E-03	-1.59
HALLMARK_INTERFERON_GAMMA_RESPONSE	3.75E-04	6.26E-03	-1.55
HALLMARK_COMPLEMENT	7.08E-04	8.85E-03	-1.51
HALLMARK_UV_RESPONSE_DN	2.56E-03	2.56E-02	-1.50
HALLMARK_INTERFERON_ALPHA_RESPONSE	5.83E-03	4.86E-02	-1.54

Supplement Table 5. Hallmarks in TINT-N ISUP 1–2 vs. TINT-D ISUP 3–5

Pathway	P value	Adj. P value	NES
HALLMARK_MYC_TARGETS_V1	1.97E-12	9.84E-11	-2.33
HALLMARK_ALLOGRAFT_REJECTION	1.30E-09	3.25E-08	-2.10
HALLMARK_INTERFERON_GAMMA_RESPONSE	1.63E-08	2.71E-07	-2.02
HALLMARK_OXIDATIVE_PHOSPHORYLATION	3.82E-05	3.82E-04	-1.66
HALLMARK_INFLAMMATORY_RESPONSE	6.39E-05	5.33E-04	-1.65
HALLMARK_PROTEIN_SECRETION	1.39E-03	5.77E-03	-1.63
HALLMARK_COMPLEMENT	9.46E-05	6.76E-04	-1.62
HALLMARK_MTORC1_SIGNALING	1.64E-04	9.10E-04	-1.59
HALLMARK_G2M_CHECKPOINT	5.51E-04	2.50E-03	-1.58
HALLMARK_KRAS_SIGNALING_UP	4.66E-04	2.33E-03	-1.55
HALLMARK_INTERFERON_ALPHA_RESPONSE	1.36E-02	4.85E-02	-1.43
HALLMARK_XENOBIOTIC_METABOLISM	5.57E-03	2.14E-02	1.47
HALLMARK_MYOGENESIS	1.57E-04	9.10E-04	1.69
HALLMARK_KRAS_SIGNALING_DN	2.09E-05	2.62E-04	1.76
