# Supplementary material for: Gene expression profiles in benign prostate tissue reflect tumor proximity, grade, and molecular subtype
Source: J Transl Med. 2026 Oct 3;24:1252. doi: 10.1186/s12967-026-09035-8 (PMC13640278; doi:10.1186/s12967-026-09035-8)
Supplement: Supplementary file 2 [file 12967_2026_9035_MOESM2_ESM.pdf]

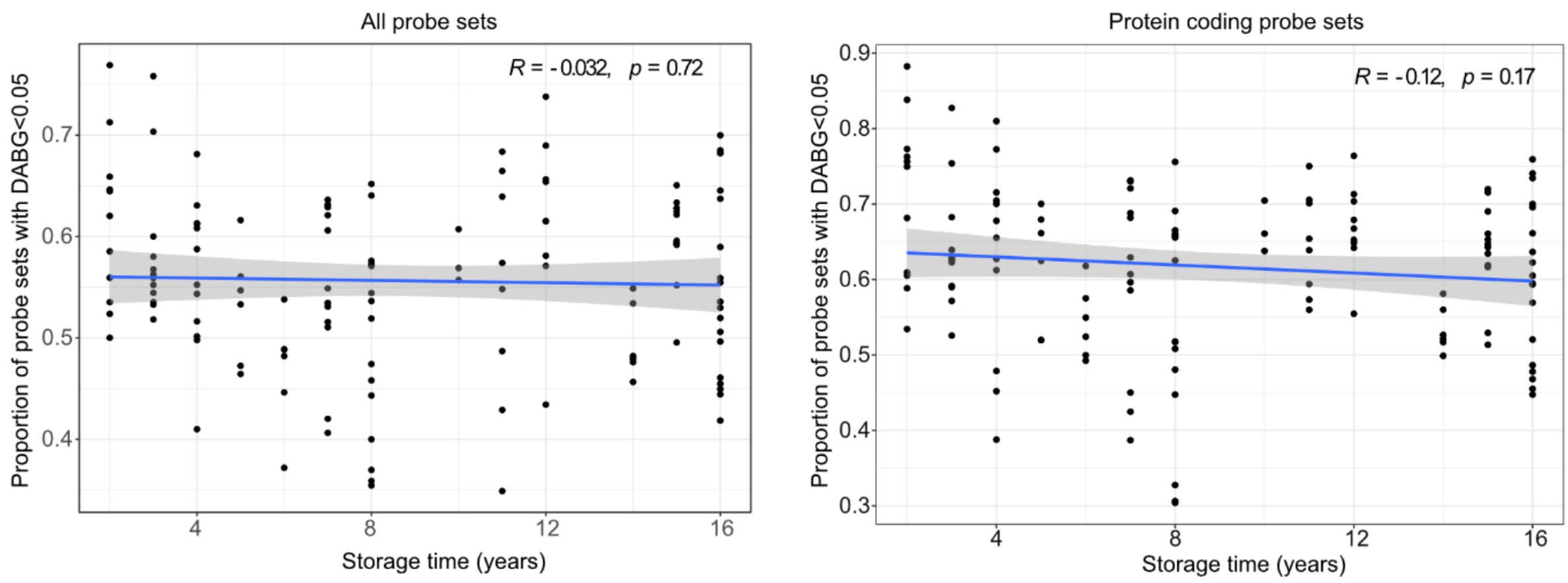

Supplement Figure 1. Association between fraction of detection and sample storage time. No associations were found between proportion of probe sets (all probe sets and protein coding probe sets) with detection above background (DABG) <0.05 and storage time of the sample paraffin blocks.

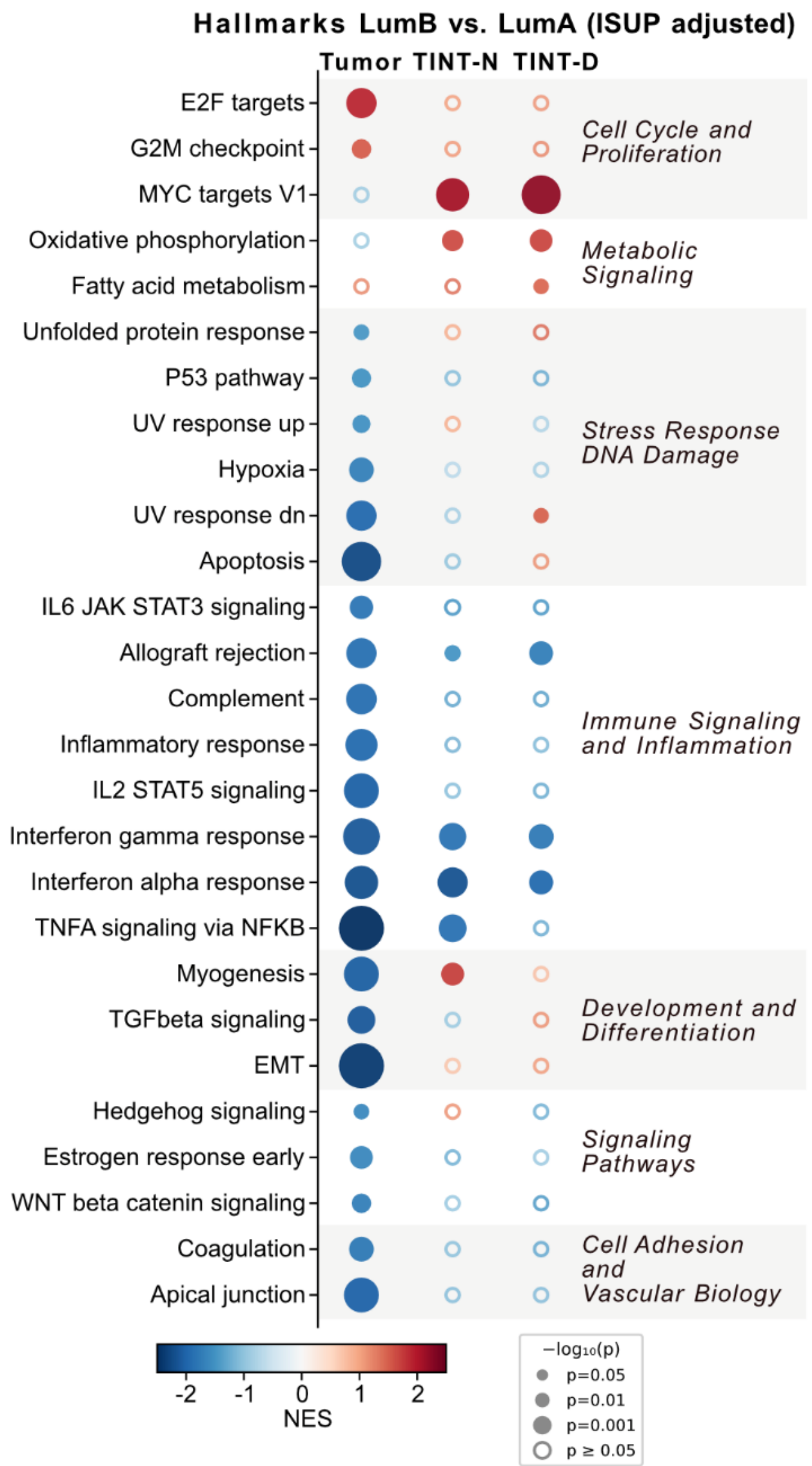

Supplement Figure 2. Gene set enrichment analysis (GSEA) of transcriptomic profiles in radical prostatectomy samples. GSEA based on mRNA profiles in radical prostatectomy tissues comparing molecular subtypes LumB (n=22) vs. LumA (n=19) adjusted for ISUP. The color scale reflects significantly induced (red) and suppressed (blue) hallmarks, according to normalized enrichment scores. Filled circles: adjusted $p < 0.05$; open circles: adjusted $p \geq 0.05$.

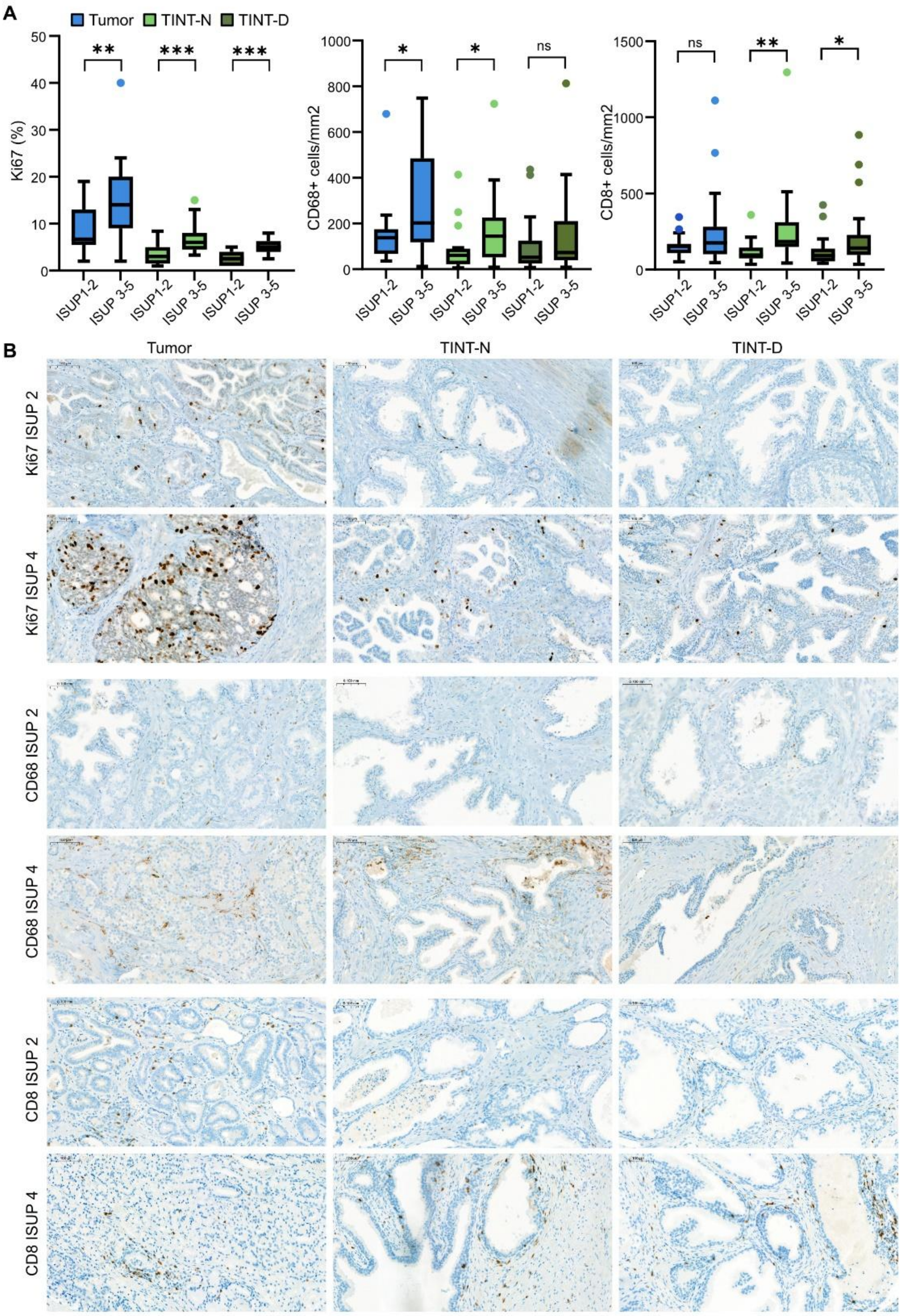

Supplement Figure 3. Quantification of Ki67, CD68, and CD8 positive cells in relation to tumor grade. A) Fraction of Ki67+ tumor cells and non-malignant prostate epithelial cells, and densities of CD68+ macrophages or CD8+ T-cells were quantified in tumor, TINT-N, and TINT-D in high-grade (ISUP 3-5, n=24) vs. low-grade (ISUP 1-2, n=20) cases. * p<0.05, **p<0.01, ns=non-significant (Mann-Whitney U) **B)** Representative sections showing Ki67, CD68, and CD8 staining in tumor, TINT-N, and TINT-D in low-grade (ISUP 2) and high-grade (ISUP 4) cases.

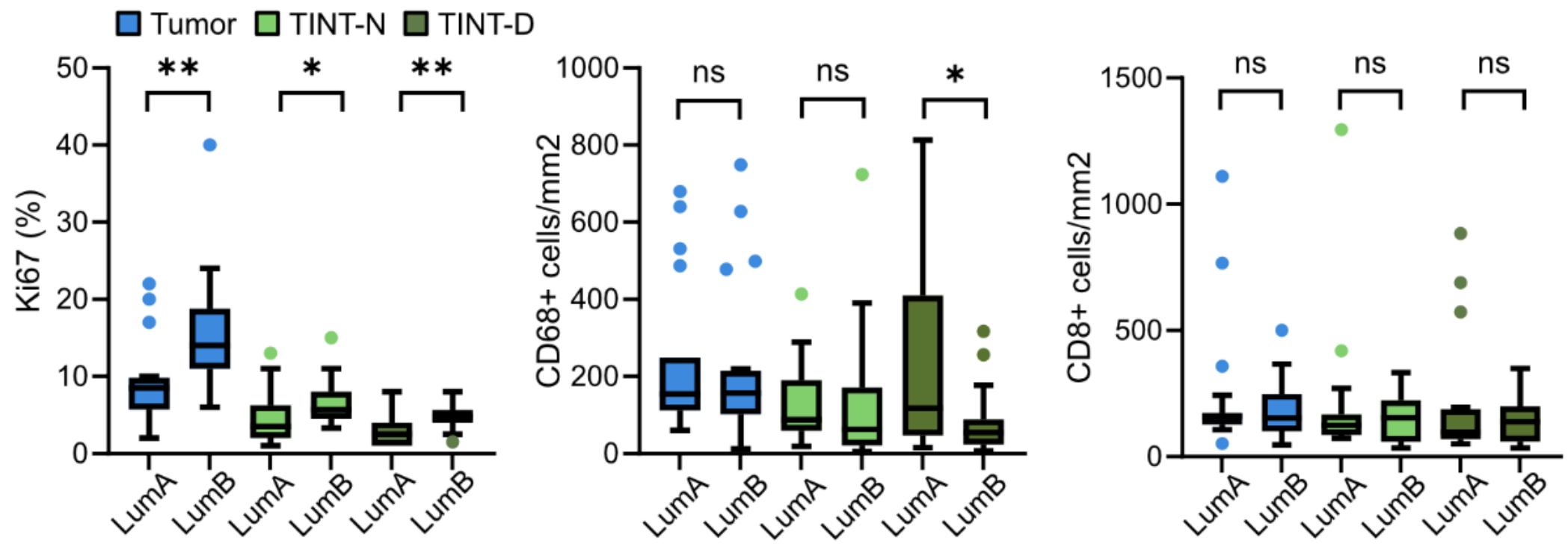

Supplement Figure 4. Quantification of Ki67, CD68, and CD8 positive cells in relation to molecular subtype. A) Fraction of Ki67+ tumor cells and non-malignant prostate epithelial cells, and densities of CD68+ macrophages or CD8+ T-cells were quantified in tumor, TINT-N, and TINT-D in Luminal B (LumB, n=22) vs. Luminal A (LumA, n=19) cases. * p<0.05, **p<0.01, ns=non-significant (Mann-Whitney U).
